# Fecal Calprotectin as a Diagnostic and Prognostic Biomarker for Gastrointestinal Graft Versus Host Disease: A Systematic Review of Literature

**DOI:** 10.7759/cureus.4143

**Published:** 2019-02-27

**Authors:** Mustafa N Malik, Abdul Rafae, Ceren Durer, Seren Durer, Faiz Anwer

**Affiliations:** 1 Internal Medicine, The University of Arizona, Tucson, USA; 2 Internal Medicine, McLaren Flint - Michigan State University, Flint, USA; 3 Hemtology and Oncology, The University of Arizona, Tucson, USA; 4 Internal Medicine, Florida Hospital, Orlando, USA; 5 Hematology and Oncology, Cleveland Clinic, Cleveland, USA

**Keywords:** biomarker, calprotectin, graft versus host disease, stem cell transplantation, diagnosis

## Abstract

The current practice for diagnosing graft versus host disease (GVHD) includes clinical or endoscopic evaluation of the patient. Clinical diagnosis is limited by an overlapping symptomatic spectrum with infectious causes, a common scenario in the post-transplant setting where an invasive procedure, such as endoscopy, is often impractical. We, therefore, evaluated the role of fecal calprotectin as a diagnostic as well as a prognostic biomarker for gastrointestinal GVHD (GI-GVHD) occurrence and severity in the post-hematopoietic transplant population. Following Prisma guidelines, we performed a systematic search of articles published after 2004 using the PubMed, Embase, Cochrane Library, and Web of Science databases. After a detailed screening, 10 studies involving a total of 494 patients were included. In the cohorts comparing median fecal calprotectin (mFC) level in GI-GVHD vs. non-GI-GVHD patients, the results indicated an increase in the mFC level in patients with GI-GVHD when compared to non-GI-GVHD patients. Similarly, an increase in the mFC level was seen in accordance with the severity of the disease. Moreover, corticosteroid-resistant patients had a higher mFC level as compared to corticosteroid-sensitive patients. Our study indicates that the mFC level can be used for diagnosing as well as predicting the treatment response to GI-GVHD. However, future randomized prospective trials involving larger populations are needed to further explore its significance.

## Introduction and background

Graft versus host disease (GVHD) is the most important complication after allogeneic hematopoietic stem cell transplantation (HSCT) and a leading cause of morbidity and mortality [1-2]. The incidence of acute gastrointestinal graft versus host disease (GI-GVHD) in HSCT patients is reported to between 40% and 50% [3]. The gold standard for the diagnosis of GVHD is an endoscopic biopsy, which is an invasive, time-consuming procedure, has the disadvantage of causing discomfort to patients, and is not always feasible due to the poor general condition of the patient [3-4]. Several biomarkers, such as serum albumin, thrombomodulin, angiopoietin-2, vascular endothelial growth factor, and fecal calprotectin, have been studied in the setting of GVHD [5].

Calprotectin is a calcium-containing protein that accounts for approximately 60% of the total proteins of the cytosol in neutrophils and macrophages. Fecal calprotectin, a heterodimer of two S100 family proteins (A8 and A9), is secreted by activated macrophages and neutrophils and acts by specifically binding to endothelial cells, thereby inducing leukocyte recruitment and the secretion of pro-inflammatory cytokines [6]. It has antibacterial and antifungal activities and induces apoptosis upon neutrophilic activation in cell cultures. Calprotectin is released upon cell disruption and cell death. Its level in feces is found to be six times higher than in plasma, and it remains stable in stool for more than seven days at a different temperature, thereby showing resistance to proteolysis [2-3,6].

Fecal calprotectin has an established role as a diagnostic modality and a biomarker of disease activity in patients with inflammatory bowel disease (IBD), with sensitivity and specificity ranging from 80%-90% [1-2,6]. It has been suggested that fecal calprotectin may also play an important role in diagnosing and assessing the severity of GVHD and in determining response and resistance to corticosteroid treatment [1-3,7]. It might also be possible to develop an approach for detecting acute GVHD before the onset of clinical manifestations [8].

The main aim of our analysis is to study the published literature on the role of fecal calprotectin as a diagnostic and prognostic biomarker in patients with GI-GVHD after allogeneic HSCT. Our secondary aim is to determine the specificity of fecal calprotectin in GI-GVHD by comparing its level with non-GI-GVHD and various etiologies of infectious diarrhea after allogeneic HSCT.

## Review

Materials and methods

**Figure 1:**
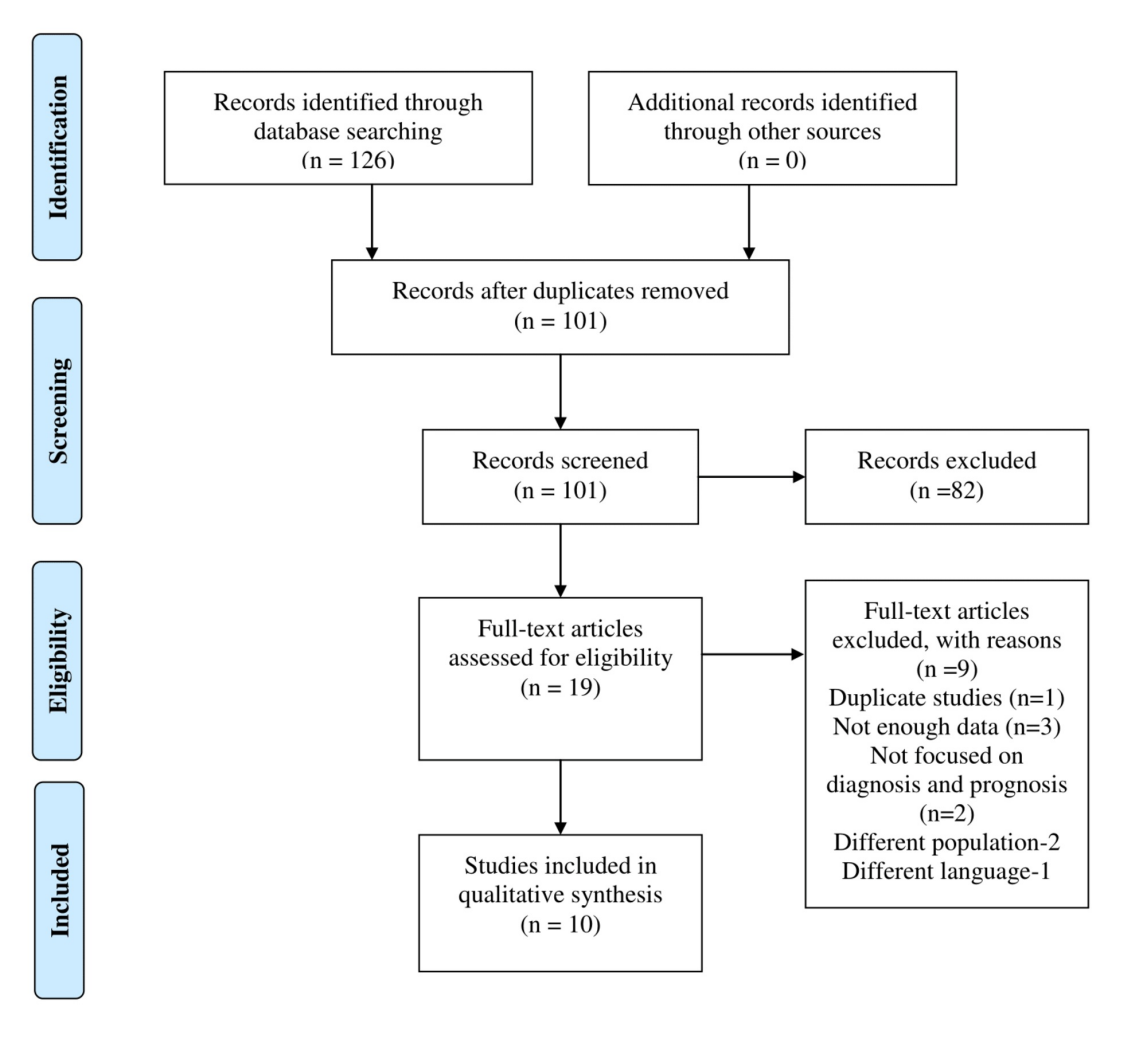
Data identification, screening, eligibility testing, and inclusion according to PRISMA guidelines PRISMA: preferred reporting items for systematic reviews and meta-analyses

Following preferred reporting items for systematic reviews and meta-analyses (PRISMA) guidelines (Figure 1), we performed a systematic search on articles published after 2004 using the PubMed/Medline, Elsevier/Embase, Wiley/Cochrane Library, and Thomas Reuters/Web of Science databases. Search results were not limited to any geographical area. Studies fulfilling the following criteria were included: (1) studies comparing median fecal calprotectin (mFC) level in GVHD and non-GVHD patients; (2) studies comparing the mFC level in GI-GVHD patients, non-GI-GVHD patients, and other infectious causes of diarrhea in post-transplant setting; (3) studies correlating the mFC level with the severity of GVHD; and (4) studies investigating fecal calprotectin in the monitoring of treatment response and treatment resistance. We included only studies in the English language. Ten studies involving a total of 494 patients were included.

One author (MNM) extracted the data, which were subsequently examined by the second author (AR). We analyzed the following variables: author, year, total number of subjects, total number, and mFC level in patients with GVHD, without GVHD, GI-GVHD, non-GI-GVHD and CMV colitis, mFC level in grade I, II, III, and IV GVHD, mFC level at treatment onset and at one week. If the desired data were not reported in a study, we documented it as not specified (NS). We summarized numeric data and categorical data using medians, ranges, and absolute values.

Results

A total of 494 patients were included. Two hundred fifty two patients (51%) developed acute GVHD. One-hundred seventy-nine patients (71%) had GI-GVHD and 73 patients (29%) had non GI-GVHD. In one of the cohorts (n=21), median fecal calprotectin (mFC) levels were 198.9 mg/kg (range(r) =58.4-500) in patients with GVHD versus 32.2 mg/kg (r=15.6-89) in patients without GVHD (p=0.0005) [6]. In a similar cohort (n=23), mFC levels were 504 mg/kg in patients with GVHD and 107.4 mg/kg in patients without GVHD (p=<0.001) [7]. Five cohorts compared the mFC level in GI-GVHD vs. non GI-GVHD patients and the results were as follows: 318 mg/kg (r=36-596) vs. 38 mg/kg (r=35.5-56), p=0.003 (61 patients)[2]; 595 mg/kg vs. 51.7 mg/kg, p=<0.001 (64 patients)[4]; 396.6 mg/kg (r=142.1-500) vs. 115.2 mg/kg (r=58.4-292.3), p=0.02 (14 patients) [6]; 500 mg/kg vs. 95 mg/kg, p=0.0229 [9]; 56 mg/kg vs. 121 mg/kg, p=0.67 (31 patients) [10]. In another cohort (n=40), the mFC level was 193.9 mg/kg in patients with GI-GVHD as compared to the mFC level of 53.1 mg/kg in patients with CMV colitis (p=0.017) [4]. In another cohort (n=14), the mFC level was 134.9 mg/kg (r=58.4-292.3) in patients with grade I-II GVHD as compared to the mFC level of 396.6 mg/kg (95.2-500) in patients with grades III-IV GVHD (p=0.029) (Table 1) [6].

**Table 1:** Fecal calprotectin levels in GVHD, GI-GVHD, and CMV colitis Abbreviations: CMV: cytomegalovirus; GVHD: graft vs. host disease; GI-GVHD: gastrointestinal graft vs. host disease; kg: kilogram; mFC: median fecal calprotectin; mg: milligram; n: number of patients; N.S: not specified; Ref.: references

Author	Year	Study Design	n	mFC (mg/kg) Level	mFC (mg/kg) Level	p-value	Ref.
GVHD vs. No GVHD				GVHD	No GVHD		
Metafuni E	2017	Retrospective Cohort	21	198.9 (58.4-500)	32.2 (15.6-89)	0.0005	[6]
Bastos Oreiro M	2012	Prospective Cohort	23	504	107.4	<0.001	[7]
GI-GVHD vs. Non GI-GVHD				GI-GVHD	Non-GI GVHD		
O'Meara A	2015	Prospective Cohort	61	318 (36-596)	38 (35.5-56)	0.003	[2]
Adam B	2016	Prospective Cohort	64	595	51.7	<0.001	[4]
Metafuni E	2017	Retrospective Cohort	14	396.6 (142.1-500)	115.2 (58.4-292.3)	0.02	[6]
Chiusolo P	2012	Prospective Cohort	N.S	500	95	0.0229	[9]
Broglie L	2018	Prospective Cohort	31	56	121	0.67	[10]
GI-GVHD vs. CMV Colitis				GI-GVHD	CMV Colitis		
Adam B	2016	Prospective Cohort	40	193.9	53.1	0.017	[4]
Predictor of GVHD Severity				Grade 1-2 GVHD	Grade 3-4 GVHD		
Metafuni E	2017	Retrospective Cohort	14	134.9 (58.4-292.3)	369.6 (95.2-500)	0.029	[6]
Lorenz F	2015	Prospective Cohort	51	204 (Grade 2)	731 (Grade 3) 2422.5 (Grade 4)	N.S	[3]

In another cohort (n=54) with GI-GVHD, corticosteroid-sensitive patients had an mFC level of 64 mg/kg (range 17-360) at onset and 49 mg/kg (range 12-238) at one week of treatment while corticosteroid-resistant patients had an mFC level of 488 mg/kg (range 65-835) at onset and 542 mg/kg (range 121-1080) at one week of treatment (p=0.028 at onset and p=0.038 at one week) [2]. In another cohort (n=7), the mean fecal calprotectin level was 24 mg/kg (r=16-31) in steroid-sensitive patients as compared to the mean fecal calprotectin level of 449 mg/kg (r=116-1111) in steroid-resistant patients (p=0.032) (Table 2) [10].

**Table 2:** Fecal calprotectin as a predictor of treatment response and steroid resistance Abbreviations: kg: kilogram; mFC: median fecal calprotectin; mg: milligram; n: number of patients; Ref.: references

Author	Year	Study Design	n	mFC at onset (mg/kg) (Steroid Responsive)	mFC at onset (mg/kg) (Steroid Resistant)	p-value	mFC at 1 week (mg/kg) (Steroid Responsive)	mFC at 1 week (mg/kg) (Steroid Resistant)	p-value	Ref.
O'Meara A.	2015	Prospective Cohort	54	64	488	0.028	49	542	0.038	[2]
Broglie L.	2018	Prospective Cohort	7	24	449	0.032				[10]
Rodriguez P.	2012	Prospective Cohort	63	<100	>100	.0001				[1]

Discussion

Allogeneic hematopoietic stem cell transplantation (HSCT) is performed for curing a wide range of hematologic malignancies [1,4,6]. Abdominal symptoms, particularly diarrhea, frequently occur after allogeneic HSCT. The main causes of diarrhea include drug-induced side effects, mucosal damage due to conditioning therapy, infection by cytomegalovirus (CMV), Epstein Barr virus (EBV), various fungi, and graft vs. host disease (GVHD) [3-4,7]. Among these, GVHD is the most important complication after allogeneic HSCT and is defined as a complex immune response by donor T cells due to the recognition of minor genetic disparities in the recipient [2]. The incidence of GVHD is 50% to 70% and it is an important cause of morbidity and mortality after allogeneic HSCT [1]. Acute GVHD is a systemic disease that can manifest in the skin, liver, and gastrointestinal tract either sequentially, simultaneously in the skin and gut, or in a single organ. GVHD is divided into four grades on the basis of Glucksberg criteria [2]. Gastrointestinal GVHD (GI-GVHD) is included in the erosive mucosal gut disease family with an incidence of 40% to 50% in patients receiving allogeneic grafts. Classical symptoms include anorexia, nausea, vomiting, abdominal pain, and diarrhea. Severe cases may result in intestinal bleeding due to mucosal ulceration and often carry a poor prognosis. Endoscopic findings range from normal mucosa to extensive edema, mucosal sloughing, and diffuse bleeding. The histological hallmark of GI-GVHD is epithelial apoptotic cell death followed by loss of crypts [3].

Endoscopic biopsy, although the gold standard test for the diagnosis of GI-GVHD, is often impractical in these patients because of overlapping infectious etiologies, which are common after allogeneic HSCT, where performing an invasive procedure is often risky. Therefore, the role of non-invasive tests can be of crucial benefit in these patients. To date, albumin, vascular endothelial growth factor, thrombomodulin, and fecal calprotectin have been studied in GVHD patients [5]. Only a few markers predict the severity of GVHD at disease onset [1-2]. Virtually no biomarkers are available to predict responses to treatment, though 30% to 50% of patients will not respond to corticosteroids [1]. Recently, fecal calprotectin has been gaining importance and it has been suggested that fecal calprotectin levels, although not very specific, may play an important role as a screening tool as well as in predicting severity and response to corticosteroids in GI-GVHD [1-3,7]. Some researchers have also commented on the possibility of detecting GVHD with fecal calprotectin even before the emergence of clinical manifestations, however, significant data are lacking [8].

Measurement of fecal calprotectin levels has an established role as a diagnostic modality and marker of disease activity in patients with inflammatory bowel disease (IBD). It has successfully been able to predict treatment response and the probability of relapse in these patients [1,6]. Fecal calprotectin levels reflect mucosal intestinal inflammation of any origin whether infectious or noninfectious. Fecal calprotectin levels depend on the migration of neutrophils into the intestinal lumen [1,3,8]. The sensitivity and specificity of fecal calprotectin to discriminate an IBD from other non-inflammatory bowel diseases range from 80% to 95%, with a usual threshold of between 50 and 100 µg/g stool [2].

Recently published literature on fecal calprotectin in GVHD has shown promising results. Metafuni et al. and Bastos Oreiro et al. have reported a statistically significant association of fecal calprotectin levels with GVHD in their studies and highlighted its role as a promising biomarker in the screening of patients with acute GVHD [6-7]. O’Meara et al., in his prospective cohort study, reported that fecal calprotectin levels were significantly elevated in patients with GI-GVHD when compared to the patients with non-GI-GVHD and the data was statistically significant [2]. Adam et al., Metafuni et al., and Chiusolo et al. have reported similar results [4,6,9]. However, Broglie et al., in his prospective cohort study, has shown conflicting results with no statistical significance [10]. The above-mentioned studies clearly reflect that fecal calprotectin can be employed specifically for GI-GVHD rather than GVHD only.

Among patients with diarrhea, higher fecal calprotectin levels distinguished between GI-GVHD and infectious enteritis following HSCT or nonspecific diarrhea after autologous stem cell transplant. Chiusolo et al., in his prospective cohort study, reported that fecal calprotectin levels were significantly lower in patients with infective colitis when compared to patients with GI-GVHD [9]. Adam et al. found a similar correlation of fecal calprotectin levels with GI-GVHD when compared to patients with CMV colitis [4]. This correlation of fecal calprotectin with GI-GVHD is promising since it can clearly distinguish patients who have gastrointestinal symptoms due to GI-GVHD from the patients who experience similar symptoms from the other infectious causes.

A positive association was demonstrated between fecal calprotectin and severity of GI-GVHD as reported by Metafuni et al. and Lorenz et al. [3,6]. In a retrospective cohort study by Metafuni et al., patients with grades 3-4 GI-GVHD had significantly higher levels of fecal calprotectin when compared to patients with grades 1-2 GI-GVHD [6]. Lorenz et al., in his prospective cohort study, have reported similar results with an increase in fecal calprotectin levels along with the severity of GI-GVHD [3]. Lorenz et al. also reported a statistically significant correlation between high fecal calprotectin and endoscopic and histological findings in GI-GVHD and it was suggested that fecal calprotectin can also be used as a predictive marker for histological findings in GI-GVHD as well [3].

The evolution of acute GVHD during the initial weeks defines the severity of GVHD and the absence of response to corticosteroids [1]. Thus, early diagnosis and timely initiation of corticosteroid therapy play a vital role in the survival of the patient [4]. First-line treatment for acute GVHD is corticosteroids but only 40% to 70% of patients respond to this treatment. Patients with steroid-resistant acute GVHD (SR-GVHD) have a very poor outcome, with long-term survival not exceeding 30% [1, 2]. There is no clearly established second-line treatment for SR-GVHD, however, immunosuppressants, mono or polyclonal antibodies, interventions like mesenchymal stem cell administration, and extracorporeal photophoresis have been tried with no improvement in long-term survival due to infectious complications [2].

Sequential monitoring of fecal calprotectin levels in patients with GVHD can indicate treatment response to corticosteroids. Moreover, fecal calprotectin levels at the onset of GVHD can help in predicting the treatment resistance. In a study by O’Meara et al., treatment-responsive patients have shown a decrease in their fecal calprotectin levels at one week of treatment. Furthermore, fecal calprotectin levels at onset and at one week after starting the treatment were significantly higher in corticosteroid-resistant patients when compared to corticosteroid responders [2]. Broglie et al. and Rodriguez et al. have reported similar results with higher fecal calprotectin levels in patients with corticosteroid resistance [1,10].

However, several studies included in our literature review were underpowered due to a small patient population, suggesting that larger randomized prospective studies are needed to validate the use of fecal calprotectin as a screening tool for the diagnosis of GI-GVHD after allogeneic stem cell transplantation for identifying those patients who are most likely to benefit from endoscopy and biopsy.

## Conclusions

In our study, we report statistically significant data on the role of fecal calprotectin in differentiating patients with GVHD, specifically GI-GVHD, when compared to patients with non-GI-GVHD and infective colitis. A linear correlation between fecal calprotectin and severity of GVHD was found. Statistically significant data on the role of fecal calprotectin in monitoring treatment response was also observed. In a nutshell, fecal calprotectin has a significant role as a biomarker in the diagnosis and prognosis of GI-GVHD. This can enable us to replace endoscopic biopsies, an invasive procedure that is currently being used for the same purpose, and can expedite the diagnosis and management of patients with GVHD, thereby improving their long-term survival. Data on fecal calprotectin levels seem promising and future randomized prospective trials are needed to further explore its role.
